# C7a, a Biphosphinic Cyclopalladated Compound, Efficiently Controls the Development of a Patient-Derived Xenograft Model of Adult T Cell Leukemia/Lymphoma

**DOI:** 10.3390/v3071041

**Published:** 2011-07-05

**Authors:** Ana B. Guimaraes-Correa, Lindsey B. Crawford, Carlos R. Figueiredo, Karina P. Gimenes, Lorena A. Pinto, Maria Fernanda Rios Grassi, Gerold Feuer, Luiz R. Travassos, Antonio C.F. Caires, Elaine G. Rodrigues, Susan J. Marriott

**Affiliations:** 1 Unidade de Oncologia Experimental, Departamento de Microbiologia, Imunologia e Parasitologia, Universidade Federal de São Paulo (UNIFESP-EPM), São Paulo 04023-062, Brazil; E-Mails: anabeatrizguima@gmail.com (A.B.G.-C.); rogernty@hotmail.com (C.R.F.); kpgimenes@yahoo.com.br (K.P.G.); travassos@unifesp.br (L.R.T.); rodrigues.elaine@unifesp.br (E.G.R.); 2 Department of Microbiology and Immunology, SUNY Upstate Medical University, Syracuse, NY 13210, USA; E-Mail: crawfoli@upstate.edu (L.B.C.); 3 Laboratorio Avançado de Saúde Pública, CPQGM, Fundação Oswaldo Cruz (FIOCRUZ), Salvador, Bahia 40296-700, Brazil; E-Mails: lorena@aluno.bahia.fiocruz.br (L.A.P.); grassi@bahia.fiocruz.br (M.F.R.G.); 4 Centro Interdisciplinar de Investigação Bioquímica, Universidade de Mogi de Cruzes, Mogi das Cruzes, São Paulo 08780-911, Brazil; E-Mail: caires@umc.br (A.C.F.C.); 5 Humurine Technologies, Inc., 640 Arrow Highway, La Verne, CA 91750, USA; E-Mail: Gerhumurine@gmail.com (G.F.); 6 Department of Molecular Virology and Microbiology, Baylor College of Medicine, Houston, Texas, TX 77030, USA

**Keywords:** cyclopalladated compound, HTLV-1, ATLL, chemotherapy, xenograft model, apoptosis

## Abstract

Adult T-cell leukemia/lymphoma (ATLL) is a highly aggressive disease that occurs in individuals infected with the human T lymphotropic virus type 1 (HTLV-1). Patients with aggressive ATLL have a poor prognosis because the leukemic cells are resistant to conventional chemotherapy. We have investigated the therapeutic efficacy of a biphosphinic cyclopalladated complex {Pd_2_ [*S_(−)_*C^2^, N-dmpa]_2_ (μ-dppe)Cl_2_}, termed C7a, in a patient-derived xenograft model of ATLL, and investigated the mechanism of C7a action in HTLV-1-positive and negative transformed T cell lines *in vitro. In vivo* survival studies in immunocompromised mice inoculated with human RV-ATL cells and intraperitoneally treated with C7a led to significantly increased survival of the treated mice. We investigated the mechanism of C7a activity *in vitro* and found that it induced mitochondrial release of cytochrome c, caspase activation, nuclear condensation and DNA degradation. These results suggest that C7a triggers apoptotic cell death in both HTLV-1 infected and uninfected human transformed T-cell lines. Significantly, C7a was not cytotoxic to peripheral blood mononuclear cells (PBMC) from healthy donors and HTLV-1-infected individuals. C7a inhibited more than 60% of the *ex vivo* spontaneous proliferation of PBMC from HTLV-1-infected individuals. These results support a potential therapeutic role for C7a in both ATLL and HTLV-1-negative T-cell lymphomas.

## 1. Introduction

About 44,000 new cases of leukemia are expected in the United States in 2011. Nearly half of these will be acute leukemias, including adult T-cell leukemia/lymphoma (ATLL). ATLL is a highly aggressive disease characterized by the rapid and uncontrolled clonal proliferation of mature transformed CD25^+^CD4^+^ T-cells. Infection with human T lymphotropic virus type 1 (HTLV-1) is the major risk factor for the development of ATLL. Currently, approximately 15 million people in the world are infected with HTLV-1. Conventional chemotherapy or a combination of zidovudine (AZT) and interferon-alpha are the currently favored treatments for patients with ATLL, but are effective in only 50% of cases [1]. Additionally, the mean survival of patients in the acute phase of ATLL is approximately six months even with aggressive chemotherapy. Although targeting HTLV-1-infected cells for apoptosis is an attractive approach to treating ATLL, HTLV-1 infected cells are generally resistant to apoptosis induced by available drugs. Thus, it is important to develop new approaches to treat HTLV-1-infected patients diagnosed with ATLL and to specifically induce cell death in HTLV-1-infected T-cells *in vivo*.

Novel antitumor compounds that are cytotoxic to tumor cells *in vitro* must be tested in preclinical animal models to determine their availability and effectiveness *in vivo*. In the absence of a syngeneic murine model for ATLL, xenogeneic experimental models have been developed, using immunocompromised mice. Mice homozygous for the SCID mutation lack functional T and B lymphocytes, fail to generate humoral and cell-mediated immunity and have been widely used as hosts for both normal and malignant human cells. Engraftment of SCID mice with patient-derived tumor cells provides an *in vivo* model in which to investigate the tumorigenic potential of HTLV-1-infected human lymphocytes and cell lines. Feuer and colleagues initially reported the development of lymphoma in C.B.-17 scid/scid mice inoculated with peripheral blood mononuclear cells (PBMC) from an ATLL patient [2,3]. This patient-derived ATLL line, termed RV-ATL, can be propagated and expanded in SCID mice and is a useful platform to test the efficacy of novel therapeutic treatments on ATLL. Further refinement of the SCID mouse model by removing the interleukin (IL)-2 receptor generated NOD.Cg-*Prkdc*^scid^ *Il2rg^tm1Wjl^*/SzJ (NSG) mice. NSG mice lack functional NK cells and are deficient in cytokine signaling, which supports better engraftment of human hematopoietic stem cells and PBMCs [4,5]. These mice also support the propagation and expansion of RV-ATL-cells.

Recently, several palladium (Pd) complexes have been evaluated as antitumor agents [6,7], but only a few of them have been tested in preclinical animal models. Cyclization of Pd complexes by cyclometallation reactions not only increased the stability but also produced less toxic complexes, making them promising antitumor compounds [8]. A group of biphosphinic cyclopalladated compounds, obtained from the cyclometallation agents, *N*,*N*-dimethyl-1-phenethyl-amine (dmpa), phenyl-2-pyridinyl-acetylene or 1-phenyl-3-*N*,*N*-dimethylamine-propyne and containing the biphosphinic ligand 1, ethanebis(diphenyl-phosphine) (dppe), were synthesized and tested *in vitro* and *in vivo* in a syngeneic murine melanoma B16F10-Nex2 model. One complex, [Pd(C2,N-(S(-) dmpa)(dppe)].Cl, named C7a, was cytotoxic to murine B16F10 melanoma cells *in vitro* at concentrations lower than 1.25 μM, and was the most active *in vivo*, delaying subcutaneous tumor growth and increasing animal survival [9]. More recently, we demonstrated that C7a reduced the number of pulmonary nodules in the murine metastatic melanoma model, and interacted with mitochondrial membrane thiol-groups to induce the intrinsic apoptotic death pathway in murine and cisplatin-resistant human tumor cells [10]. Hebeler-Barbosa *et al.* demonstrated the additive anti-melanoma protective effect of C7a in a gene therapy protocol with plasmids encoding IL-12 and an Fc-chimera of the soluble α-chain of IL-13 receptor. The combined therapy significantly reduced the subcutaneous tumor evolution with 30% tumor-free mice [11].

The present study investigates the effect of C7a in an ATLL mouse model and the mechanism of cell death induced by C7a in HTLV-1-infected and uninfected T cell leukemia lines. We show that treatment with C7a significantly increased the survival of RV-ATL engrafted mice and that C7a induced caspase-mediated apoptosis of human transformed T-cell lines and HTLV-1 infected T cells. These results support a potential therapeutic role for C7a in both ATLL and HTLV-1-negative T-cell lymphomas.

## 2. Results

### 2.1. C7a Is Cytotoxic to Human Leukemia T Cell Lines

**Figure 1:**
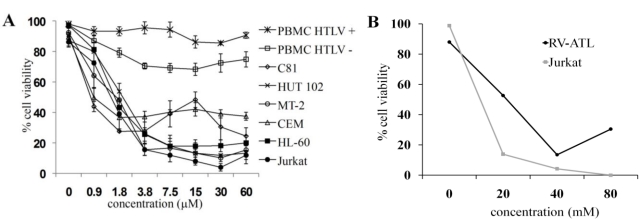
Cyclopalladated C7a is cytotoxic to human leukemia cell lines, but has little effect on human T lymphotropic virus type 1 (HTLV-1)-infected or uninfected peripheral blood mononuclear cells (PBMC). (**A**) 5 × 10^5^ PBMC (previously stimulated with PHA and IL-2) from three donors (HTLV-1 uninfected), 10^5^ cells from human T cell leukemia lines, and 10^5^ PBMC from four different HTLV-1-infected individuals were seeded in 96-well plates in triplicate. Cells were incubated with concentrations of C7a as indicated for 48 h, and cytotoxicity was determined by Trypan blue exclusion in an automated cell counter. The bars represent the means and SD of three different experiments; (**B**) RV-ATL cells were expanded in a SCID mouse for three weeks. Freshly harvested cells were seeded at 10^5^ cells per well in a 24-well plate. The cells were incubated with concentrations of C7a as indicated for 48 h. Uninfected human Jurkat T cells were used as a control and incubated in the same conditions. Cytotoxicity was determined by Trypan blue exclusion and manual counting. A representative experiment is shown. The experiment was repeated twice, using two independent frozen tissue culture stocks of RV-ATL cells and without refreshing the media or drug, with comparable results. In (A) and (B), the viability of treated cells was expressed as percent of control untreated cells.

Because HTLV-1 infected transformed T cells are difficult to control with available chemotherapeutic strategies, we evaluated the cytotoxic effect of C7a on HTLV infected cell lines *in vitro*. C7a was cytotoxic to all cell lines in a dose-dependent manner (Figure 1A) suggesting that established human T cell lines are susceptible to C7a whether or not they are infected with HTLV-1. Although C7a did not kill 100% of any of the cell lines analyzed, even at the highest concentration tested, 7.5 μM C7a reduced the viability of most cell lines by 90% after 48 h. Uninfected CEM T cells were most resistant to C7a cytotoxicity, with 40% of the cells remaining viable at 60 μM C7a. Importantly, C7a had minimal cytotoxic effect on the viability of HTLV-1 infected or uninfected primary human PBMC, with >80% of the cells remaining viable even at 60 μM C7a (Figure 1A). In a similar dose-dependent cytotoxicity study to examine the cytotoxicity of C7a, 40 μM C7a reduced the viability of RV-ATL cells, a patient-derived HTLV-1-infected ATLL cell line, by 90% after 48 h (Figure 1B).

### 2.2. C7a Is Effective in a Preclinical Model of ATLL

Since C7a could kill RV-ATL cells *in vitro*, we next evaluated the effect of C7a on the development of leukemia *in vivo* using a xenogeneic murine model [2,3]. RV-ATL cells (10^7^), previously expanded *in vivo* in NOD.Cg-*Prkdc^scid^ Il2rg^tm1Wjl^*/SzJ (NSG) mice, were intraperitoneally inoculated on day 0 in 23-week old NSG mice. The engraftment of mice with RV-ATL cells was monitored by peritoneal lavage at days 14, 28 and 42. Collected cells were analyzed by flow cytometry for previously determined phenotypic markers and were huCD45^+^, huCD4^+^, huCD8^−^ and huCD25^low^ (data not shown). All mice were successfully engrafted.

**Figure 2:**
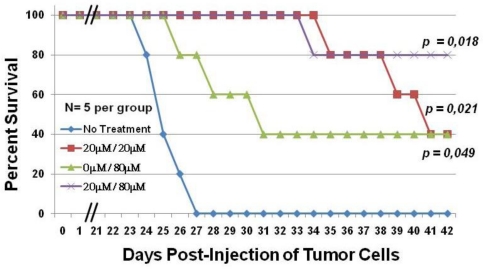
C7a significantly increases the survival of RV-ATL tumor bearing mice. Adult female NSG mice were intraperitoneally (IP) injected with 10^7^ fresh, *in vivo* expanded RV-ATL cells on Day 0. Mice were treated with either C7a (20 μM diluted in PBS) (red line and purple line) or PBS alone (blue line and green line) by IP injection every other day starting on Day 4. Starting on day 22, half the mice from each group were treated with 80 μM C7a (green line and purple line). N = 5 per group for four treatment groups. All mice were treated by IP injections through day 40, and survival was monitored daily. *p* values (Kaplan Meier test) are shown for each group, compared to no treatment group.

NSG mice engrafted with RV-ATL cells were divided into four groups (five mice per group). Each group of mice was inoculated with the same dose of C7a diluted in 100 μL of PBS, regardless of body weight, and drug concentration per gram of mouse body weight was not statistically different based on weekly weight measurements (data not shown). All mice were monitored daily for clinical assessment and survival. Animals in group 1, which did not receive any C7a, had an average survival of 24 days post-inoculation (Figure 2), and none of the mice survived beyond day 27 (blue line). Animals in group 2, which received 20 μM C7a every other day beginning on day 4 through day 40, survived significantly longer than animals in group 1 (*p* value = 0.049), with all animals surviving to day 34 and two animals still alive at termination of the study (red line). Animals in group 3 did not receive C7a until day 22, which allowed tumor expansion prior to treatment. These animals then received 80 μM C7a every other day from day 22 through day 40 (green line). Animals in group 3 survived longer than animals in group 1 that did not receive any C7a. Although three animals did not survive past day 31, the remaining two animals in group 3 were still alive at termination of the study suggesting that a higher dose of C7a late in tumor development can protect a significant fraction of animals.

Animals in group 4 received 20 μM C7a every other day from day 4 to day 20, and 80 μM C7a every other day from day 22 to day 40 (purple line). These animals showed the best survival, with four of the five animals in this group still alive at termination of the study. Pairwise comparison of the treatment groups showed that the survival of animals in group 4 was significantly better than those in group 1 (*p* value = 0.018). In all groups, animal deaths were attributed to tumor burden. Clinical signs prior to death included distended abdomen due to tumor growth, weight loss, dragging of hind limbs (necropsy showed tumor pressing on thigh muscles and/or spinal cord). There was no evidence of clinical signs that were not tumor related.

### 2.3. C7a Induces the Intrinsic Apoptotic Pathway in Human T Cell Leukemia Lines

**Figure 3:**
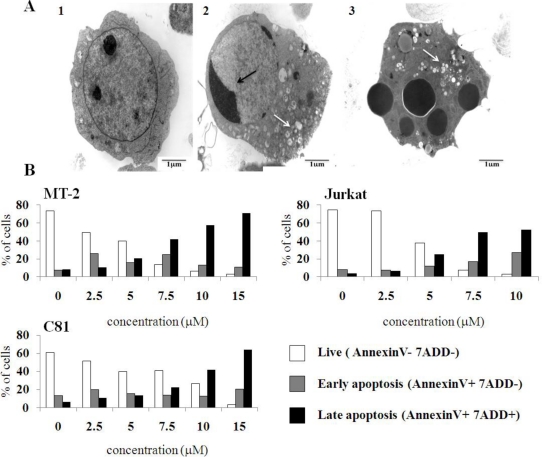
C7a induces morphological and phenotypic alterations compatible with apoptosis in human leukemia cell lines. (**A**) Transmission electron microscopy. (1) HL-60 cells cultivated in complete RPMI medium for 6 h; (2 and 3) HL-60 cells cultivated in presence of 2 μM C7a for 7 h. Black arrows: nuclear condensation; White arrows: vacuoles. This assay was repeated twice, and representative images are shown; (**B**) Membrane phosphatidylserine expression. HTLV-1 infected MT-2 and C81, and uninfected Jurkat cell lines (1 × 10^6^) were incubated in the presence of C7a at the indicated concentrations for 3 h at 37 °C. After staining with PE-Annexin V and 7-AAD, cells were analyzed by flow cytometry, as described in Material and Methods. A representative of two independent experiments is shown.

Treatment of HL-60 cells, which were effectively killed by 7.5 μM C7a (Figure 1), with 2 μM C7a for 7 h resulted in profound morphologic alterations observed by transmission electron microscopy. Treated cells showed chromatin margination, and an increase in enlarged vacuoles and electron-dense material in the cytoplasm (Figure 3A). Externalization of phosphatidylserine (PS), which is normally restricted to the inner leaflet of the plasma membrane, is a hallmark of mammalian apoptosis. Cell lines were treated with C7a for 3 h, and PS exposure was analyzed by double staining the cells with 7-amino-actinomycin (7-ADD), a vital dye, and PE-annexin V, a Ca^2+^ dependent phospholipid-binding protein that has a high affinity for PS (Figure 3B). A dose-dependent decrease in the number of live cells (PE-annexin negative and 7-ADD negative) and an increase in the number of cells in late apoptosis (PE-annexin positive and 7-ADD positive) were observed in MT-2, C81, and Jurkat cell lines treated with C7a for 3 h (Figure 3B). The morphological changes, and the PS exposure observed in C7a-treated T cell leukemia lines suggested that this cyclopalladated complex can induce apoptosis in these cells, as observed previously for murine melanoma cells.

**Figure 4:**
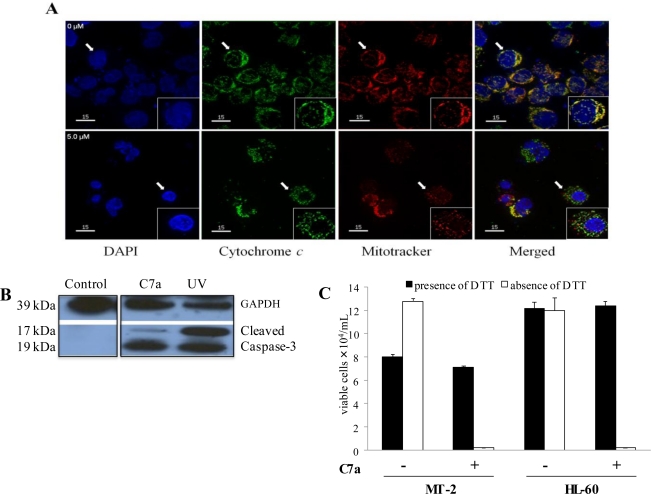
Mitochondria are involved in C7a induced cell death. (**A**) Cytochrome c release. HTLV-1 infected MT-2 cells were incubated without (0 μM) or with 5 μM C7a for 3 h. The cells were fixed and incubated with anti-cytochrome *c* antibody (green). Mitochondria were detected by MitoTracker staining (red), and nuclei were visualized with DAPI (blue). Magnification (×40). Inserts: Single cell indicated by the arrow, magnification (×60). Rare cells treated with C7a that show colocalization of cytochrome c and mitochondria likely reflect the short 3 h exposure to C7a. The assay was repeated twice, and representative images are shown; (**B**) Caspase Activation. Cell lysates from MT-2 cells before treatment (Control), after treatment with 5 μM C7a for 3 h, or after exposure to 50 J of UV light (UV), as a positive control, were analyzed by Western blotting for cleaved caspase-3 and GAPDH as a loading control. This assay was repeated twice, and representative images are shown; (**C**) DTT inhibits C7a effect on leukemic human T cells. MT-2 (HTLV-1 infected) and uninfected HL-60 cells (10^5^) were pre-incubated or not with 1 mM of DTT for 1 h. C7a was added to the culture (15 μM to MT-2, 2 μM to HL-60 cells) and incubated for 6 h. Viable cells were counted in presence of Trypan Blue in a hemocytometer. The bars represent the means and SD of three different experiments.

To determine the effect of C7a on mitochondria of intact HTLV-1-infected T cell leukemia lines, MT-2 cells were incubated *in vitro* with 5 μM C7a for 3 h. In untreated cells, cytochrome c colocalized with the mitochondrial marker (top row, see white arrow) (Figure 4A). In contrast, in cells treated with C7a, cytochrome c was predominately localized in the cytoplasm and did not colocalize with the mitochondrial marker (bottom rows). These results suggest that C7a can induce mitochondrial permeabilization and release of mitochondrial contents.

C7a-induced apoptosis was associated with caspase activation in MT-2 cells, as the caspase-3 precursor was cleaved into the 17 and 19 kDa death-associated fragments (Figure 4B). C7a similarly induced activation of caspase 3 in HL-60 cells as well as activated caspases -8 and -9 (data not shown). Although DTT showed a partial toxicity to MT-2 cells, C7a -mediated cytotoxicity was abolished by DTT treatment in both cell lines (Figure 4C), suggesting that this cyclopalladated complex can induce thiol-cross-linking of mitochondrial membrane proteins resulting in mitochondrial permeabilization in leukemia cells, as previously described in murine melanoma cells treated with the *R*(*+*) enantiomer of C7a [12].

To investigate whether the lysosomal pathway plays a role in apoptotic cell death induced in leukemia cells by C7a, the cytotoxic effect of C7a was evaluated in MT-2 and HL-60 cells after preincubating with a cathepsin B inhibitor (CA-074). The cytotoxic effect of C7a was not influenced by the inhibitor, indicating that the lysosomal pathway is not involved in C7a-mediated apoptosis of malignant T cells (data not shown).

**Figure 5:**
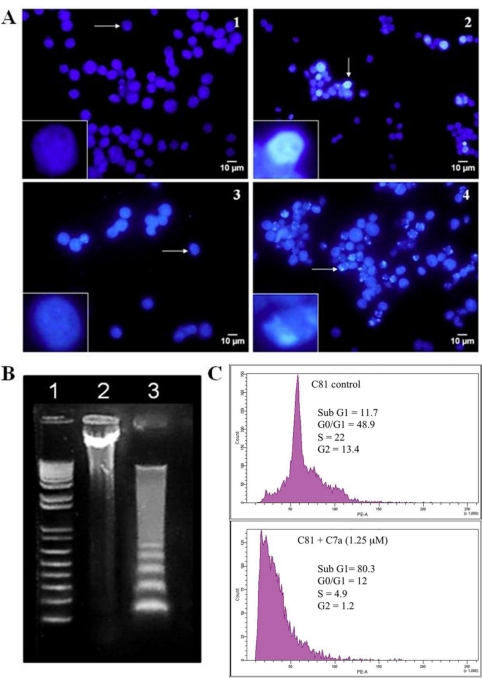
C7a induces nuclear alterations characteristic of apoptosis. (**A**) Nuclear condensation. Uninfected HL-60 cells were untreated (1) or treated with 5 μM C7a (2) for 6 h. Alternatively, HL-60 cells were untreated (3) or treated with 2.5 μM C7a (4) for 24 h. After incubation with C7a, cell nuclei were stained with Hoechst 33342. Inserts: Single cell indicated by the arrow, ×25 magnification. The assay was repeated three times, and representative images are shown; (**B**) DNA fragmentation. HL-60 cells (3 × 10^6^) were treated (lane 3) with 5 μM of C7a or not (lane 2) for 18 h. Lane 1: MW standard. The assay was repeated three times, and a representative gel is shown; (**C**) Cell cycle analysis. HTLV-1 infected C81 cells were cultured with 1.25 μM C7a (bottom panel) or not (top panel) for 48 h. Cells were stained with propidium iodide (50 μg/mL) and cell cycle analysis was performed by flow cytometry. Percentage of cells in each phase of the cycle is shown.

Nuclear effects of C7a were evaluated in human leukemia T cell lines. Nuclear condensation was observed in HL-60 cells treated with 5 μM C7a for 6 h (Figure 5A, panel 2) and chromosomal fragmentation was observed after 24 h (Figure 5A, panel 4), while few cells showed such patterns in control untreated cells (Figure 5A, panels 1 and 3). After treatment with 5 μM C7a for 18 h, HL-60 cells showed DNA internucleosomal fragmentation (Figure 5B, lane 3). Upon propidium iodide staining, fluorescence intensity in FACS-analyzed cells varies linearly with DNA content, ranging from 2 n (G0-G1 phases) to 4 n (G2 phase) with an intermediate plateau that corresponds to S phase. The sub-G1 peak in DNA histograms corresponds to the apoptotic population. Exposure of HTLV-1 infected C81 cells to 1.25 μM C7a for 48 h induced an 8-fold increase in the sub-G1 peak (Figure 5C, bottom panel), again demonstrating that C7a induces apoptosis in these cells. Taken together, these results show that C7a induces the intrinsic apoptotic pathway in human leukemic T cell lines, with no participation of the lysosomal pathway.

### 2.4. C7a Treatment Reduces Spontaneous Proliferation of PBMC from HTLV-1-Infected Patients

**Figure 6:**
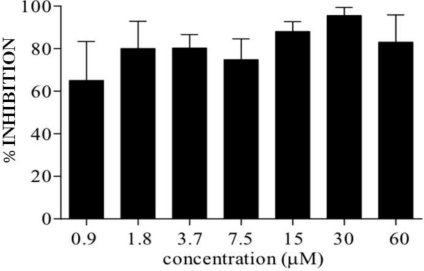
*In vitro* inhibition of spontaneous proliferation of PBMC from HTLV-1-infected patients by C7a. Isolated PBMC from HTLV-1-infected individuals were cultured in the presence or absence of C7a compound for 24 h. Bars represent average inhibition and standard deviation from four patients, compared to cells from the same patient cultivated in the absence of C7a.

C7a does not have a cytotoxic effect on PBMC from healthy HTLV-1-infected individuals (Figure 1A). However, C7a was able to reduce the spontaneous *in vitro* proliferation of PBMC from HTLV-1-infected individuals (Figure 6). Spontaneous proliferation of HTLV-1-infected PBMCs was reduced to 60% after 24 h incubation with the lowest C7a concentration evaluated (0.9 μM) and reached 90% with 30 and 60 μM concentrations of C7a.

## 3. Discussion

Recently, a number of new compounds and therapies have been shown to specifically induce apoptosis in HTLV-1 infected cells and in ATLL leukemic cells [13]. Here we have demonstrated that RV-ATL engrafted mice treated with C7a, a biphosphinic cyclopalladated compound, demonstrated up to 80% survival over the course of the study, with significantly greater protection against tumor-mediated death than untreated mice. In addition, we demonstrated that C7a effectively induced mitochondria-dependent apoptosis in human HTLV-1-infected T cells. Interestingly, C7a also induced the same effects in human transformed cell lines that are not HTLV-1-infected, such as acute T-lymphoblastic leukemia HL-60, CEM and Jurkat.

Although important progress has been made in the treatment of ATLL such as combination therapy with AZT and IFN-α [14], immunotherapy with anti-IL-2 receptor or anti-CC chemokine receptor 4 monoclonal antibodies [15], allogeneic bone marrow or stem cell transplantation [16] and apoptosis induction [17–21], ATLL remains highly intractable to conventional therapeutics. Testing of novel compounds against cancer *in vivo* requires the use of preclinical animal models. Establishment of newer strains of immunocompromised mice capable of supporting efficient levels of engraftment of ATLL cells from patients has accelerated the testing of drugs and targeted therapy against ATLL [22]. To test the antitumor effects of C7a in an *in vivo* model of ATLL, we used an established ATLL patient-derived xenograft model in immunocompromised (NSG) mice [2,3]. NSG mice injected intraperitoneally with RV-ATL cells develop lymphoma associated with the mesenteric and pancreatic lymph nodes, liver and spleen. Significantly, mice treated with low dose C7a (20 μM) for the duration of the study had a 35% greater survival than untreated mice. Interestingly, mice that were untreated for the first half of the study and started on high dose (80 μM) C7a at day 22 (only two days prior to the average death of untreated mice) survived longer than untreated mice. Forty percent of these mice survived for the duration of the study suggesting that C7a is also effective against established tumors.

Over the last 15 years studies showed that arsenic trioxide (As_2_O_3_), alone or in combination with other pro-apoptotic stimuli, induces apoptosis in HTLV-1-infected cells lines [23,24] and have been examined as possible treatments for ATLL. As_2_O_3_ induces apoptosis leading to cytochrome c release and caspase activation [25]. Previous studies demonstrated that a combination of As_2_O_3_ and IFN-γ, known to trigger Tax proteolysis, cures Tax-driven ATLL in mice [26]. However clinical use of arsenic is problematic as there are differences in sensitivity to As_2_O_3_, and arsenic itself is toxic at high doses [13]. C7a and the *R*(*+*) enantiomer complex have been described as potent inducers of apoptosis through targeting of the mitochondria by interacting with thiol-groups in the mitochondrial membrane of murine melanoma cells [10], and in rat isolated mitochondria [12], respectively. Treatment of isolated rat liver mitochondria with the *R*(*+*) enantiomer of C7a, {Pd_2_ [*R*_(_*_+_*_)_C^2^, N-dmpa]_2_ (μ-dppe)Cl_2_} results in mitochondrial permeabilization as indicated by Ca^+2^- and ROS-independent mitochondrial swelling, release of cytochrome *c*, dissipation of the mitochondrial transmembrane potential, uncoupling of oxidative phosphorylation, and mitochondrial calcium release [12]. Additionally, an ionic cyclopalladated compound with a ferrocene ligand {[Pd(C2,*N*-(S(-) dmpa)(dppf)] Cl}] (termed BPC) induces apoptosis in acute leukemia cells through a novel lysosomal pathway with cathepsin B acting as the death mediator [27]. The C7a complex used in this study induces mitochondrial-dependent, lysosomal-independent, apoptosis in HTLV-1-infected and uninfected transformed cell lines as characterized by the cleavage of caspase 3 and release of cytochrome c. Thus, while cyclopalladated compounds have been clearly demonstrated to induce apoptosis by activating a variety of cellular pathways, C7a is the only compound that has demonstrated antitumor effects *in vivo* [9].

A hallmark of HTLV-1-infection is the spontaneous proliferation of peripheral blood mononuclear cells (PBMC) that is observed in approximately 50% of HTLV-1-infected individuals [28]. We found that C7a could inhibit more than 80% of the spontaneous proliferation of PBMC from HTLV-1-infected individuals, the majority diagnosed with HAM/TSP disease. The mechanisms involved in the inhibition of spontaneous proliferation by C7a remains unclear and further studies are necessary to elucidate the mechanisms of action of this drug on cell proliferation.

Since C7a is capable of inducing mitochondrial-dependent apoptosis in acute leukemia cells (HL-60), and peripheral T-cell lymphomas have a low response rate to anthracyclin-based combination chemotherapy and high relapse rate [29], C7a offers an exciting alternative for treating other peripheral lymphomas. Further studies to test the efficacy of C7a in *in vivo* leukemia models are required.

## 4. Experimental Section

### 4.1. Culture Conditions for Human Leukemia Cell Lineages and PBMC Isolation

Peripheral blood mononuclear cells (PBMC) from heparinized venous blood of three healthy controls were isolated by Ficoll-Hypaque density gradient centrifugation (Pharmacia Biotech, Uppsala, Sweden). PBMC were *in vitro* stimulated for 3 days with 2 μg/mL phytohaemagglutinin (PHA) and subsequently cultured with 10 U/mL human recombinant IL-2 (both from Sigma-Aldrich, St. Louis, MO, USA) for 2 more days. PBMC from one asymptomatic HTLV-infected individual and seven patients diagnosed with HAM/TSP were obtained from heparinized venous blood samples by SepCell density gradient centrifugation (LGCBiotechnology, São Paulo, Brazil). Samples were screened for HTLV-1/2 antibodies by an enzyme-linked immunosorbent assay (ELISA) (Ab-Capture ELISA test system; Ortho-Clinical Diagnostics, Inc., Raritan, New Jersey, USA), and results were confirmed by Western blotting assay (HTLV Blot 2.4; Genelabs Technologies, Singapore). All patients presented high proviral load (>5000 copies/10^6^ PBMCs). The Ethical Board of Oswaldo Cruz Foundation (FIOCRUZ) approved this study, and informed consent was obtained from all enrolled patients. Immortalized human cell lines used in this study were HTLV-infected T cell lines (C81, HUT 102 and MT-2) and non-infected T cell leukemia lineages (CEM, HL-60 and Jurkat). Cell lines as well as PBMC were cultured *in vitro* in complete RPMI medium (RPMI 1640 medium supplemented with 2 mM L-glutamine, 1% non-essential amino acids, 1mM sodium pyruvate, 100U/ml penicillin, 100 μg/mL streptomycin and 10% fetal bovine serum, all provided by Sigma-Aldrich). PBMC from HTLV-infected patients were cultured *in vitro* in RPMI 1640 medium supplemented with 5% human group AB serum.

RV-ATL cells were isolated from an ATL patient and expanded in SCID mice as previously described [2,3]. The RV-ATL cell line was further modified by the addition of a luciferase cassette [30]. The full phenotype of the RV-ATL cell line is human CD45^+^, CD4^+^, CD8^low^, CD25^low^ [2]. This tumor line was originally derived from PBL from a patient with acute leukemia. The phenotype of the RV-ATL cell line used in this study is human CD45^+^, CD4^+^, CD25^low^, which is comparable to both the primary derived RV-ATL cell line and to the malignant lymphocytes from the original patient. CD25^low^ expression is a reflection of the specific patient from which these cells were derived. This RV-ATL cell line is the same malignant clone derived from the original patient sample as molecularly determined by Southern blot analysis of HTLV integration. RV-ATL cells were cultivated for short term *in vitro* experiments in IMDM medium supplemented with 10% fetal bovine serum and 100 U/mL penicillin/streptomycin/glutamine.

#### Cyclopalladated compound

The cyclopalladated complex C7a was synthesized from *N,N*-dimethyl-1-phenethylamide (dmpa), complexed to 1,2 ethanebis (diphenylphosphine, dppe) ligand, as previously described in [9]. The compound was diluted to a final concentration of 10 mM in DMSO (cell culture tested, Sigma Aldrich), and for *in vivo* and *in vitro* assays diluted to the final concentration in media or PBS.

#### *In vitro* cellular toxicity assay

To evaluate the cytotoxic effects of C7a on PBMC (HTLV-1 positive and HTLV-1 negative individuals) and in malignant human T cell lineages, 1 or 5 × 10^5^ cells were seeded in 96-well plates in triplicate, serial dilutions of C7a (ranging from 0.9 to 60 μM) were added and plates were incubated for 48 h. Cellular viability was measured by Trypan Blue exclusion dye using the automated cell counter Vi-Cell XR Viability Analyzer (Beckman Coulter^®^). Control cells were incubated in the absence of C7a, and considered to be 100% viable. RV-ATL cells were expanded by intraperitoneal inoculation of SCID mice for 3 weeks and freshly harvested cells were plated at 10^5^ cells per well in a 24-well plate. For additional studies, RV-ATL cells were thawed from frozen stocks, plated in IMDM with 10% FBS and 1% P/S/G for 6 h at 37 °C prior to treatment with C7a. All RV-ATL cells were incubated in the presence of C7a at concentrations between 0 and 80 μM, diluted in complete culture IMDM media at 37 °C for 48 h. Cytotoxicity was determined by Trypan blue method in a hemocytometer. C7a compound was diluted in complete culture media, and plates were incubated at 37 °C in a 5% CO_2_ humidified atmosphere. To verify the inhibitory effect of dithiothreitol (DTT) or a lysosomal cathepsin B inhibitor (CA-074) on C7a cytotoxicity *in vitro*, 10^5^ MT-2 or HL-60 cells were pre-incubated with 1 mM DTT for 1 hour or with 10 mM CA-074 [*N*-(l-3-*trans*-propylcarbamoyl-oxirane-2-carbonyl)-l-isoleucyl-l-proline] for 2 h (both from Sigma Aldrich). C7a was added to MT-2 (15 μM) and HL-60 cells (2 μM) and incubated for 6 (DTT) or 18 h (CA-074). Viable cells were counted by Trypan Blue exclusion in a hemocytometer. Assays were done in triplicate, and repeated three times.

#### RV-ATL SCID Mouse Studies

The animal research described in this manuscript was performed according to the guidelines for the Committee for Humane Use of Animals at SUNY Upstate Medical University in accordance with all federal, state and local guidelines. NOD.Cg-*Prkdc^scid^ Il2rg^tm1Wjl^*/SzJ (NSG) mice were obtained from our own breeding colony (breeder mice originally from Jackson Laboratories). All mice were housed under specific pathogen-free conditions. Prior to all manipulations, mice were anesthetized with isofluorane. RV-ATL cells from frozen stocks were expanded intraperitoneally in NSG mice prior to injection of study mice. Age-matched (22.5 to 23 week old) female NSG mice were intraperitoneally (IP) injected with 10^7^ RV-ATL cells on day 0. Mice were treated with 100 μL of PBS (with or without C7a) by IP injection every other day from day 4 through 42. Drug concentration per gram of mouse body weight was not statistically different based on weekly weight measurements (data not shown). Tumor cells were removed by peritoneal lavage using PBS at two-week intervals (study days 14, 28 and 42). Mice were euthanized by carbon dioxide exposure as necessary due to tumor burden or severe clinical symptoms (usually dehydration). Remaining mice were euthanized on day 42 upon termination of the experiment.

#### Transmission Electron Microscopy analysis

For transmission electron microscopy (TEM) analysis, HL-60 cells (5 × 10^6^) were cultured in presence or absence of 2 μM C7a for 6 h in complete RPMI medium. Cells were fixed in 2.5% glutaraldehyde in 0.1 M sodium cacodylate buffer, pH 7.4, and post-fixation was performed using 1% osmium tetroxide, 0.08% potassium ferricyanide, 5 mM calcium chloride in the same buffer for 60 min in the dark (all reagents from Sigma-Aldrich). Cells were dehydrated using an acetone series and infiltrated in polybed epoxy resin (Polysciences, PA, USA). Ultrathin sections obtained by ultramicrotomy were collected on 300 mesh grids and stained with uranyl acetate and lead citrate for contrast. The ultrastructure analysis was done in a transmission electron microscope (Zeiss EM-900).

#### Cytochrome c release assay

MT-2 cells, cultured in round coverslips (24 mm), were preincubated for 30 min with 62.5 nM MitoTracker (Molecular Probes, Eugene, OR) and then treated with 5 μM of C7a for 3 h. The cells were washed once with PEM buffer [80 mM piperazine-*N,N′-*bis(2-ethanesulfonic) acid pH 6.8, 5 mM EGTA pH 7.0, 2 mM MgCl_2_], and fixed for 30 min at 4 °C with 5% formaldehyde diluted in PEM buffer. Cells were washed three times in PEM buffer, and permeabilized for 30 min at room temperature in PEM buffer containing 0.5% Triton X-100. Anti-cytochrome c antibody (1:800, Cell Signaling, MA, USA) diluted in 5% BSA and 0.1% Tween 20-containing TBS (TBS-T) was incubated overnight at 4 °C. After washing three times in TBS-T, cells were incubated for 2 h at room temperature in the dark with a fluorescein isothiocyanate-conjugated anti-rabbit IgG Alexa 488 (1:400, Sigma-Aldrich, MO USA) diluted in 5% BSA-containing TBS-T. Coverslips were washed three times with TBS-T, cells were counter-stained with DAPI (Sigma-Aldrich) and mounted on slides using Slow-Fade Anti-fade Mounting Media (Molecular Probes). Deconvolution images were taken using Olympus IX71 with 60X oil objective (NA = 1.42). All images were deconvolved using Delta Vision software (Applied Precision) using Ratio (conservative) method, with medium noise filtering for 6 cycles and correction applied. Single staining images were processed by the ACDsee software (ACD Systems, Canada).

#### Detection of caspase-3 activation

MT-2 cells were treated or not with 5 μM of C7a for 1, 2 or 3 h. Alternatively, cells were exposed to 50J of UV light for 5 seconds as a positive control. Cell extracts were prepared as described in [31] and separated by electrophoresis in 0.1% SDS, 12% polyacrylamide gels. Proteins were blotted onto nitrocellulose membranes, and subsequently blocked for 1h at room temperature with 5% BSA in TBS-T. Membranes were incubated with an antibody against cleaved caspase-3 (CellSignaling, MA, USA**)**, and GAPDH as a load control (Sigma-Aldrich MO, USA), diluted 1:1000 in TBS-T, 5% skim milk), overnight at 4 °C. After washing with TBS-T, the membranes were incubated with anti-rabbit Ig conjugated to horseradish peroxidase (Sigma Aldrich, diluted 1: 40000 in TBS-T, 5% skim milk). After 1 h incubation at room temperature, the membranes were washed extensively with TBS-T, and developed with ECL (GE Healthcare) according to the manufacturer’s instructions.

### 4.2. Analysis of Nuclear Alterations

#### Hoescht 33342 staining:

HL-60 cells (1 × 10^6^) were plated in 12 wells plate and treated or not (control) with 2.5 μM C7a for 6 h or 5 μM for 24 h. Cells were then collected, washed 3 times in PBS and collected on round coverslips by centrifugation at 3000 rpm for 10 min. Cells were fixed for 15 min in 2% formaldehyde and 30 min in absolute methanol, washed and stained with Hoescht 33342 (Sigma-Aldrich MO, USA) for 15 min. Coverslips were mounted on glass slides with Vectashield (4 μL), sealed with nail polish and immediately analyzed in a fluorescent Olympus BX61 microscope (magnification 400X) at 360 nm. The images were acquired using Cell^M Software.

#### DNA degradation assay:

HL-60 cells (3 × 10^6^) were incubated with 5 μM C7a at 37 °C for 18 h in 6 well-plates. Cells were recovered from the culture supernatant by centrifugation, lysed in TELT buffer (50 mM Tris-HCl pH 8.0, Triton X-100 0.4%, 2.5 mM EDTA pH 9.0 and 2.5M LiCl), and centrifuged for 15 min at 12000 g/4 °C. DNA was extracted by phenol-chloroform and precipitated with 0.1 volume of 3 M sodium acetate, pH 7.0, and 2.5 volumes of absolute ethanol, following incubation at −80 °C for 20 min. All reagents were obtained from Sigma-Aldrich. Precipitated DNA was centrifuged, diluted in RNase-free water (25 μg/mL), separated by electrophoresis on a 1% agarose gel (100V) and photographed with a digital camera (Kodak, EDAS DC290).

### 4.3. Flow Cytometry Analysis

#### Cell-cycle analysis

C81, Jurkat, HL-60 and MT-2 cells (1 × 10^6^) were cultured in the absence or presence of 2.5 μM C7a for 48 h in 24 well-plates at 37 °C in a 5% CO_2_ humidified atmosphere. Cells were then collected, fixed in chilled methanol, and suspended in solution containing RNase A (100 U/mL; Sigma Aldrich) before staining with 50 μg/mL of propidium iodide. A minimum of 1 × 10^5^ cells were acquired by flow cytometry in a FACSCanto and data was analyzed using the FACSDiva software (Becton Dickinson, Mountain View, CA).

#### Assessment of tumor burden in RV-ATL engrafted mice

Tumor burden in RV-ATL engrafted mice was assessed by flow cytometric analysis of peritoneal lavage cells. Engrafted cells were harvested by peritoneal lavage, washed with PBS and blocked in PBS containing 5% each human and murine serum for at least 20 min at 4 °C. RV-ATL cells were identified by staining with antibodies for human CD45, human CD4 and human CD25 compared with murine CD45 (all antibodies from Biolegend) for at least 30 min at 4 °C. Cells were washed twice with PBS and fixed for 15 min at 4 °C in 2% formalin in water then acquired on an LSRII flow cytometer (Becton Dickinson) and analyzed by FlowJo software (TreeStar, Ashland, CA).

#### Detection of phosphatidylserine translocation by PE-Annexin V/7-AAD

C81, Jurkat and MT-2 cells were incubated in the presence of C7a at concentrations ranging from 0 to 15 μM at 37 °C for 3 h. Untreated and treated cells were then stained with PE-Annexin V and 7-AAD (7-Amino-actinomycin D), using the Apoptosis Detection Kit I (BD Pharmingen, NJ, USA) according to the manufacturer’s instructions. The cells were acquired by flow cytometry (LSRFortessa, Becton Dickinson) and analyzed by FACSDiva software.

#### *In vitro* HTLV-1-infected PBMC proliferation assay

To evaluate the effects of C7a on spontaneous *in vitro* proliferation of HTLV-1-infected PBMC, PBMC from one asymptomatic HTLV-infected individual and six patients diagnosed with HAM/TSP were cultured separately in 96-well U-bottom culture plates (Costar, Cambridge, MA, 1 × 10^5^ cells/well), in triplicate, at 37 °C in a 5% CO_2_ humidified atmosphere. Serial dilutions (0.9 to 60 μM) of C7a and 5 μM CarboxyFluorescein diacetate Succinimidyl Ester (CFSE, Invitrogen, Eugene, USA) were added to the cells, and after 24 h incubation, cells were harvested, washed two times in 2 mL of PBS containing 1% bovine serum albumin, and fixed in PBS containing 4% paraformaldehyde. Analyses were performed using a FACSAria (Becton Dickinson) and FlowJo software. At least 10^5^ events were analyzed per sample. Proliferation intensity was determined by the percentage of divided cells and the division index. The cut-off values were >0.06 for the division index and >5.8% for the percentage of divided cells (3X the division index mean and 3X the percentage of divided cells in PBMC controls.).

## 5. Conclusions

C7a suppresses proliferation of HTLV-1-infected patient PBMCs, induces apoptosis in transformed cell lines and significantly enhances the survival of ATLL tumor bearing mice. C7a is a potential novel therapeutic treatment for malignant T-cell diseases.
